# The timing of intubation and principles of ICU care in COVID-19

**DOI:** 10.3906/sag-2106-182

**Published:** 2021-12-17

**Authors:** Sema TURAN, Sultan SEVİM YAKIN, Levent YAMANEL

**Affiliations:** 1 Department of Intensive Care, Ankara City Hospital, University of Health Sciences, Ankara Turkey; 2 Department of Intensive Care, Gülhane Faculty of Medicine, University of Health Sciences, Ankara Turkey

**Keywords:** Coronavirus disease-19, intensive care unit, intubation

## Abstract

Coronavirus disease-19 (COVID-19) has been a serious health problem since it was first identified in Wuhan, China, in December 2019 and has created a global crisis with its economic, sociological, and psychological aspects. Approximately 15% of cases have a severe clinical presentation, and 5% of patients require admission to the intensive care unit. A significant proportion of patients presents with a rapidly progressing acute respiratory failure and require invasive mechanical ventilation. This article aimed to evaluate how the optimal intubation timing should be determined in cases of acute respiratory failure due to COVID-19 and to offer recommendations for basic intensive care support in the light of our current knowledge.

## 1. Introduction

Coronavirus disease-19 (COVID-19) has been a serious health problem since it was first identified in Wuhan, China, in December 2019 and has created a global crisis with its economic, sociological, and psychological aspects. World Health Organization declared this outbreak a “public health emergency of international concern” on January 31, 2020.

The clinical presentation of COVID-19 varies in severity from asymptomatic infection to severe illness. Approximately 15% of cases have a severe clinical presentation, and 5% of patients require admission to the intensive care unit (ICU) [1]. A significant proportion of patients presents with a rapidly progressing acute respiratory failure and require invasive mechanical ventilation [2]. The initially suggested approach included early intubation and mechanical ventilation with a lung-protective strategy recommended [3,4]. Since the mortality rate of invasively ventilated patients remained high, it was hypothesized that some patients with severe COVID-19 pneumonia might benefit from other oxygenation improvement strategies allowing avoidance of invasive mechanical ventilation and its adverse effects, such as ventilator-induced lung injury and ventilator-associated pneumonia [5].

This article aimed to evaluate how the optimal intubation timing should be determined in cases of acute respiratory failure due to COVID-19 and offer recommendations for basic intensive care support in the light of our current knowledge. 

## 2. Intensive care support and COVID-19

The COVID-19 pandemic has made a significant impact on international health and healthcare delivery. The rapid spread of the virus, the high numbers of cases, and the high proportion of patients requiring respiratory support have placed unprecedented demand on ICU, necessitating rapid expansion of ICU infrastructure, capacity, and staffing in many countries [6]. 

Li et al. summarized the critical issues related to intensive care in the pandemic process. The issues in countries were listed as the lack of intensive care bed capacities, the scarcity of trained intensive care personnel, and the disruptions experienced in the unit that should be provided to patients in need of intensive care due to non-COVID-19 reasons. They emphasized the necessity of making the appropriate organization in each city for the patients who need to be administered medical treatment and providing adequate protective equipment and experienced personnel in cases where high-risk invasive procedures are required (such as ECMO or CRRT teams). The intensive care physicians’ ability to quickly analyze and respond to new diseases and to research needs to be improved since intensive care medicine requires more cooperation for its multidisciplinary approaches. For the newly emerged diseases, it is urgent to establish a special disease database and specimen bank and to share research data from different medical centers. Moreover, it is critical to promote ICU informatization construction and establish the national remote consultation platform through 4G or 5G [7].

## 3. Basic intensive care support 

The most vital reason for the need for intensive care in COVID-19 cases is acute respiratory failure, and it ranks first with a rate of over 85%. Other reasons requiring admission to intensive care are less frequent and can be listed as shock, acute kidney failure, and cardiac causes [8]. The main target of SARS-CoV-2 infection in humans is the angiotensin-converting enzyme 2 gene. In addition to the alveoli, the angiotensin converting enzyme 2 gene is highly expressed in the heart, kidney, and small intestine, meaning that the virus may also infect these organs [9].

In COVID-19 patients, symptoms of severe respiratory infection may occur through rapidly developing acute respiratory distress syndrome and other serious complications, which may be followed eventually by multiple organ failure and death. Therefore, ICU admission, follow-up, and management of critically ill patients with COVID-19 are extremely vital, and early diagnosis and timely treatment of critical cases are crucial. The need for intensive care might differ according to institutions or even countries, depending on the demand and supply, at rates ranging from 5% to 32% [2,10].

COVID-19 may present a wide clinical spectrum including asymptomatic cases, mild upper respiratory infections, and severe pneumonia [10,11]. Some patients with COVID-19–associated acute respiratory distress syndrome (ARDS) recover within several days, while others require mechanical ventilation for weeks or fail to recover at all. The reasons regarding the discrepancy are still unknown, and it is difficult to predict an individual patient’s prognosis. 

The pathogenesis of COVID-19 remains unclear. It is thought that uncontrolled pulmonary inflammation, fluid accumulation, and progressive fibrosis play a major role in compromised oxygen and carbon dioxide exchange [12]. Furthermore, a complex immune response of the host to the SARS-CoV-2 virus may result in an uncontrolled release of inflammatory proteins [13]. 

Gattinoni et al. described two types (type L and type H) of ARDS caused by COVID-19 according to lung compliance, inflammation severity on computerized tomography and response to positive end expiratory pressure (PEEP) [14]. Following this description, many criticisms have been made. Unlike this, Bos et al. defined that COVID-19-associated ARDS has no difference with the classical ARDS. They emphasized that patients may have normal lung compliance at the beginning of the infection, and as the disease progresses, the lung compliance may decrease [15]. This condition is similar to the ARDS associated with non-COVID infectious agents. Although COVID-19-induced ARDS has been described in many different ways in the literature, the most crucial point is to provide individualized respiratory support. 

A COVID-19 patient, if there is respiratory distress, may have a respiratory rate of above 30, an increase in oxygen demand in the follow-up, PaO_2_/FiO_2_ ratio of below 300, hypotension, tachycardia, concomitant acute kidney damage, signs of acute organ dysfunctions such as impaired liver function tests, or changes in consciousness. These findings suggest that the patient requires follow-up in the ICU. Cases with skin perfusion disorders such as delay in capillary refill time or cutis marmaratus on examination, lactate elevation (>2 mmol/L) and elevation in troponin levels in laboratory tests should be primarily evaluated in terms of the need for ICU admission. Generally, respiratory failure due to COVID-19 presents as a hypoxemic respiratory failure, although to a lesser extent, hypercapnic respiratory failure may also develop.

Severe pneumonia, ARDS, sepsis, and septic shock are among the leading causes of ICU admission in patients with COVID-19. Severe pneumonia is defined as signs of respiratory tract infection such as respiratory rate above 30, dyspnea, tachypnea, use of auxiliary respiratory muscles, respiratory distress symptoms such as abdominal breathing, having oxygen saturation in room air below 90%, or PaO_2_/FiO_2_ ratio below 300 under oxygen therapy. If the respiratory distress symptoms occurred in the last 7 days and could not be explained by clinical heart failure or volume excess, the patient is considered to have ARDS. Sepsis is a clinical presentation with organ dysfunction resulting from uncontrolled inflammation response in the host against infection. It has a high mortality rate due to the development of acute organ failure findings such as respiratory failure, changes in consciousness, hyperbilirubinemia, thrombocytopenia, coagulopathy, tachycardia, hypotension, and renal failure. Septic shock is characterized by the underlying circulatory and/or metabolic disorders in a patient with sepsis severe enough to increase the mortality rate prominently. Persistent hypotension requiring vasopressor support to maintain the mean arterial pressure at 65 mmHg despite adequate fluid replacement in a patient with sepsis and a lactate level of ≥2 mmol/L (in the absence of fluid deficit) is defined as septic shock. 

Although the presentation of severe disease associated with COVID-19 is mainly severe pneumonia and acute respiratory distress syndrome reported in 60%–70% of patients, there might also be the symptoms of sepsis and septic shock (30%), myocarditis, arrhythmia, and cardiogenic shock (20%–30%), and acute kidney injury (10%–30%) [16].

The indications for ICU admission vary in many countries due to their admission criteria. In Turkey, specialists use the Turkish guidelines, as shown in the table, prepared as per COVID-19 Advisory Committee proposals (Table 1).

**Table 1:** The primary indications for ICU admission according to Turkish Scientific Committee Guidelines.

Patients with a respiratory rate of ≥30
Dyspnea and increased work of breathing
SpO2 <90% or <70 mmHg (in room air)
Oxygen requirement ≥5 L/min with nasal cannula
Lactate >2 mmol/L

## 4. Respiratory support in COVID-19 ARDS

The clinical progression from the initial symptoms to pneumonia is about 5 days, and the median time to ICU admission from the start of hypoxemia is 7–12 days [17]. Management of pneumonia and respiratory failure due to COVID-19 is the most critical step affecting mortality in these patients. Recognizing hypoxemic respiratory failure early and administering proper oxygen therapy is vital. Although oxygen could be given through high-flow nasal cannula (HFNC) and noninvasive mechanical ventilation (NIV), low-flow oxygen delivery systems can also be used, such as a nasal cannula, simple face mask, and nonrebreather masks with a reservoir or combination of them. The oxygen support method should be determined by considering the patient’s oxygen saturation, blood gas parameters, and clinical findings. Up to 6 L/min of oxygen can be administered by nasal cannula without FiO_2_ exceeding 45%. For patients who need oxygen support over 6 L/min, a simple face mask and a nonrebreathing reservoir mask should be preferred. A simple face mask is started with 5 L/min of oxygen, increasing to a maximum of 8 L/min, which provides up to 60% FiO_2_. With the nonrebreathing reservoir mask, FiO_2_ can be increased up to >85% with a flow rate of 10-15 L/min. It should not be forgotten that FiO_2_ > 60% for more than 6 h may cause oxygen toxicity, and it must be titrated according to SpO_2_. Although using these noninvasive oxygen delivery systems may reduce the need for invasive mechanical ventilation, it is thought that they may have some risks for the airborne spread of the virus. It should also be remembered that prolonged spontaneous breathing may cause patient self-inflicted lung injury, similar to ventilator-induced lung injury that may induce uncontrolled intrathoracic negative pressures and be prevented by timely decision for intubation [18] (Table 2). 

**Table 2:** Oxygen therapy methods in COVID-19–related respiratory failure.

Low-flow oxygen therapies	High-flow oxygentherapies	Noninvasive ventilation	Invasive ventilation	ECMO
24%–100% FiO2 could be provided with 1–6 L/minwith a nasal cannula 5–8 L/min with a simple mask, or 10–15 L/min with a nonrebreathing oxygen mask with a reservoir.	Provides respiratory support with up to 60 L/min and 100% FiO2.A surgical mask should be worn on the face.It is recommended to be applied in negative pressure rooms.It can be combined with prone position.	It can be applied in selected tachypneic and hypoxic patients who need positive pressure ventilation.It is recommended for use in negative pressure rooms.NIV efficiency should be closely monitored in the NIV-administered group. In case of NIV failure, there should be no delay in transition to invasive ventilation.	Endotracheal intubation and invasive ventilation are recommended in hypoxic/hypercarbic cases whose respiratory failure is not controlled by other oxygen support therapies.Lung protective ventilation and optimal PEEP support should be given.In cases with resistant hypoxia, appropriate mechanical ventilation support should be provided with neuromuscular muscle relaxant infusion under prone position and sedation.	It is recommended to be used incases with a PaO2/FiO2 ratio below 150 and under 65 years of age despite optimal mechanical ventilation therapy.Although it carries high-mortality,it can be life-saving in selected cases.Venovenous ECMO is the preferred ECMO support method used in isolated respiratory failure.

FiO2, fraction of inspired oxygen; PaO2, partial arterial oxygen pressure; ECMO, extracorporeal membrane oxygenation; NIV, noninvasive ventilation; PEEP, positive end expiratory pressure.

A recent systematic review has indicated that compared to conventional oxygen therapy, HFNC decreases the risk of requiring intubation without affecting mortality. The authors emphasized that flow rates varied between the studies, and also, duration of treatment was not analyzed. A physiologic randomized controlled study revealed that the higher (60 L/min) the flow, the better the physiologic response [19,20].

Patel et al. reported that NIV delivered by helmet reduced intubation rates in patients with ARDS more significantly than NIV delivered by a facial mask (from 61% to 18%, respectively) [21]. As the helmet seems to be a more effective and tolerable interface in this setting, it would make sense to evaluate its effect compared to HFNC [22]. Indeed, very recently, a physiological randomized cross-over study concluded that in patients with PaO_2_/FiO_2_, high-PEEP helmet NIV could be preferred over HFNC to optimize oxygenation and mitigate the inspiratory effort, especially in most severely hypoxemic patients and in those exhibiting intense inspiratory effort during HFNC. Patients with low inspiratory effort during HFNC should be followed up cautiously since they can experience increased dynamic transpulmonary driving pressure while on NIV with the helmet [23].

The most critical question in COVID-19 patients who cannot be provided adequate oxygenation and are hemodynamically unstable despite all these oxygen support treatments is when to switch to invasive ventilation. 

## 5. Deciding the optimal intubation time in a COVID-19 patient

Approximately 14%–30% of hospitalized patients with COVID-19 develop severe respiratory failure requiring ICU admission [24–26]. Endotracheal intubation rates vary significantly between studies from 3.2% to 88%, most likely due to variability in study populations, study environments, or intubation criteria [26–29]. Moreover, the intubation timing is different between patients. It was reported that most of the intubated patients required intubation within the first 2 days of hospital admission, and the duration of ventilation was reported to range from 4 to 30 days. Indications for endotracheal intubation in COVID-19 include commonly severe respiratory distress, hypoxia, and loss of consciousness [30–32]. However, a small number of patients require intubation secondary to heart failure or airway obstruction [33]. Several factors are reported to be associated with higher intubation frequency, including advanced age, obesity, male sex, and underlying systemic diseases such as hypertension and diabetes. Increased blood levels of ferritin, D-dimer, and lipase correlate with ICU admission and intubation risk [34–37]. 

At the beginning of the pandemic in China, the mortality rate of the patients who required mechanical ventilation was high as 80% [38]. Nevertheless, as the pandemic has progressed, mortality rate has decreased. Recent analysis indicates that the mortality rate ranges between 15% and 36% following intubation [39–41].

The timing of endotracheal intubation in COVID-19 patients with respiratory failure is controversial. In the early phase of the pandemic, several guidelines from China, the United Kingdom, the United States of America, and Australia recommended early intubation of patients with ARDS to protect healthcare workers from cross-infection and avoid complications associated with urgent intubations [42,43]. However, reports over time revealed high mortality rates in intubated patients and forced clinicians to delay intubation by using high-flow oxygen systems [44]. Nevertheless, it is thought that these systems may cause patient self-induced lung injury, which exacerbates further the lung injury due to intense respiratory effort in a spontaneously breathing patient, causing barotrauma and possibly vascular trauma in the already affected lungs. Clinicians believed that variability in transpulmonary and intravascular pressure could contribute to local trauma [45]. On the other hand, delayed intubation might cause death. In a retrospective cohort study of critically ill patients related to COVID-19 pneumonia, Bavishi et al. demonstrated that later intubation was associated with a higher mortality rate than early intubation, but they could not find a significant difference in parameters of lung mechanics related to worsening of ARDS [46]. Recently, some observational and retrospective studies have indicated no significant difference between early, late, or no intubation in mortality of critically ill COVID-19 patients with ARDS [47]. One of the recommendations about deciding intubation timing in COVID-related ARDS patients is the ROX index which is described as the ratio of SpO2/FiO2 to respiratory rate [48]. Roca et al. found out a correlation between ROX index and requirement for mechanical ventilation. In their study, ROX greater than or equal to 4.88 measured at 2, 6 , or 12 h after HFNC initiation was consistently associated with a lower risk for intubation. A ROX less than 2.85, less than 3.47, and less than 3.85 at 2, 6, and 12 h of HFNC initiation, respectively, were predictors of HFNC failure and requiring intubation [49]. However, there is no strong recommendation about a threshold that could be used for intubation timing, and a clinician must consider many other factors to decide intubation. The decision to intubate may be an art of medicine, yet, in times of such crisis, when doctors from different fields and with different skills, or even young doctors without specialties, are encountered in the decision making, formal thresholds and sound protocols should be introduced. The decision and timing of intubation should be decided on a case-by-case basis in COVID-19 patients. There is a need for more studies to get more information regarding the timing of intubation. 

## 6. Prone positioning

The prone position is a widely accepted method in severe ARDS. It improves the ventilation/perfusion ratio and recruitment of the dorsal lung segments, resulting in the opening of collapsed dorsal alveoli with better gas exchange and oxygenation. In awake, nonintubated, and spontaneously breathing patients with hypoxemic respiratory failure (majorly immunocompromised), prone positioning revealed a significant improvement in PaO_2_/FiO_2_ [50].

In mechanically ventilated non-COVID-19 patients with severe ARDS, those who were mechanically ventilated in prone position had a lower mortality rate [51]. However, the clinical outcomes of prone position in COVID-19 (intubated/nonintubated] patients remain unclear. Chua et al. emphasize that prone position affects oxygenation and improves PaO_2_/FiO_2_ ratio with better than supine position in COVID-19 patients [52]. The prone position expands the collapsed dorsal lung region, resulting in a better ventilation/perfusion ratio and a more homogenous distribution of lung ventilation. Regional ventilation changes in the prone and supine position can be observed in both normal and ARDS lung, indicating an even distribution of distending forces throughout the lung tissues. The distribution of pulmonary blood flow in the normal or diseased lung is mainly directed dorsally, either in the supine or prone position. With this relatively constant regional perfusion in the prone position along with a significant improvement in lung homogeneity, the effect of shunt fraction is expected to reduce and lead to a marked improvement in oxygenation. This condition demonstrated in animal and human studies that the relative shunt fraction of prone position was reduced by 30% than the supine group with injured lungs. However, Gattinoni et al. reported that the improvement in oxygenation during prone position did not persist after returning to supine position and the PaO_2_/FiO_2_ ratio returned to baseline at 6 h following re-placing in the supine position [53] . This may be explained by the recollapse of the previously opened dorsal lung units during prone position, resulting in ventilation/perfusion mismatch and rebound hypoxemia [54–59]. Early prone positioning added to HFNC or NIV avoided the need for intubation in up to half of the patients with moderate to severe ARDS, including those with viral pneumonia [60].

It is known that there are perfusion problems as well as ventilation in severe ARDS cases associated with COVID-19. This condition, called immunothrombosis, causes microthrombus formations in the pulmonary vascular bed. The shunt rate increases due to deterioration of pulmonary perfusion and contributes to the development of resistant hypoxia. Studies have revealed the benefit of adding anticoagulants to the treatment in these cases [61,62]. In severe ARDS cases, both pulmonary microvascular thrombosis and mechanical ventilation therapy [high PEEP values] may cause right heart failure. In these cases, the use of inhaled nitric oxide by echocardiographic evaluation is recommended by several studies. However, the effect of these treatments on mortality has not been demonstrated [63].

## 7. Medical treatments in intensive care unit

In COVID-19 patients admitted to the intensive care unit, the mentioned basic intensive care support should be provided, while medical treatments should be arranged simultaneously [64]. Among the medical treatments administered so far, antiviral, antiinflammatory, anticoagulant treatments are the most basic medical treatment approaches.

### 7.1 Antiviral Medication

Although almost sixteen months have passed since the beginning of the pandemic, there is still no antiviral treatment with definite effectiveness against COVID-19. However, the antiviral treatments used in COVID-19 so far can be listed as endosomal acidification inhibitors, RNA synthesis inhibitors, and protease inhibitors.

Chloroquine and hydroxychloroquine demonstrate their main antiviral activity against SARS-CoV-2 by increasing the endosomal pH and inhibiting the entry of the virus into the cell. When the results of clinical trials investigating the efficacy of hydroxychloroquine/chloroquine in the treatment of COVID-19 were evaluated, it was concluded that the viral activation pathway targeted by chloroquine and hydroxychloroquine does not work in respiratory tract cells. Therefore, it is unlikely that these agents will be effective in the treatment or prevention of the disease. One of the most critical points to be considered in patients taking hydroxychloroquine or chloroquine in intensive care is that this drug can cause QT prolongation and/or ventricular tachycardia, including torsades-de-pointes. In this case, it is important to determine whether corrected QT interval is >500 ms by daily ECG monitoring [65].

Favipiravir (T-705; 6-fluoro-3-hydroxy-2-pyrazinecarboxamide), a guanosine purine nucleotide analog, undergoes phosphoribosylation inside the cell and turns into its active form called favipiravir ribofuranosyl-5B-triphosphate (Favipiravir-RTP) Favipiravir-RTP is a potent inhibitor of RdRp but ineffective against both DNA-dependent RNA polymerase and DNA polymerase. Therefore, it is effective only against RNA viruses but not against DNA viruses and human cells. Only the oral form of favipiravir is metabolized by aldehyde oxidase [AO] in the liver, and its inactive metabolite T-705M1 is excreted by the kidneys. Although there is no clear result on the efficacy of favipiravir in the treatment of COVID-19, there is information that using an early and effective dose would be beneficial. It is recommended to continue the favipiravir treatment and administer favipravir at least 7 days when admitted to the intensive care unit. In their study, Shrestha et al. found that patients had a significant improvement in FVP groups on both the 7th and 14th days of treatment (Day 7: RR 1.25, 95% CI 1.01 to 1.53; Day 14: RR 1.29, 95% CI 1.08 to 1.54) [66].

Remdesivir is another antiviral, and its mechanism of action is thought to be the premature termination of viral RNA transcription as an adenosine nucleoside analog. Although there is no clear result regarding its efficiency against COVID-19, two critical points to be considered in intensive care in patients receiving this treatment are kidney and liver failures. Dosage adjustment is not recommended in patients with GFR ≥30 mL/min in renal failure, yet, the drug is not recommended in patients with renal failure. If AST and ALT levels are greater than five times the upper limit of the normal range as in liver failure, the treatment should not be initiated, and if it occurs during treatment, the treatment should be stopped [67]. Lopinavir and ritonavir are agents used in HIV treatment as protease inhibitors. In SARS-CoV-2, their use has not been shown to be effective on mortality, but their use in pregnant women is recommended [68].

### 7.2 Antiinflammatory therapy

COVID-19 has three consecutive stages of increasing severity. The early stage is characterized by infection with SARS-CoV-2. In this phase, flu-like symptoms can develop, mainly due to the viral infection itself. Subsequently, patients can develop viral pneumonia, requiring hospitalization or even mechanical ventilation. The second stage is also characterized by pulmonary inflammation and coagulopathy, which can develop consecutively but often overlap. In addition, increased levels of inflammatory biomarkers such as C-reactive protein [CRP], ferritin, IL-6, IL-1, and D-dimer are associated with the development of ARDS and an unfavorable clinical course. Finally, the third stage of the disease is characterized by fibrosis [69].

Antiinflammatory treatments are administered in the hyperinflammatory phase of the disease in the intensive care unit. At this stage, glucocorticoids, IL-6 antagonists, and IL-1 antagonists are used mainly. When studies investigating the effects of these drugs on mortality are evaluated, it has been shown that glucocorticoids, in particular, provide a lower 28-day mortality rate. In REACT Working Group’s newly published prospective metaanalaysis pooled data from 7 randomized clinical trials. In this metaanalaysis, treatment doses of corticosteroids were 15 mg/day of dexamethasone, 400 mg/day of hydrocortisone, and 1 mg/kg/day of methylprednisolone. The fixed-effect summary OR for the association with mortality was 0.64 (95% CI, 0.50–0.82; P < 0.001) for dexamethasone compared with usual care or placebo (3 trials, 1282 patients, and 527 deaths), the OR was 0.69 (95% CI, 0.43–1.12; P = 0.13) for hydrocortisone (3 trials, 374 patients, and 94 deaths), and the OR was 0.91 (95% CI, 0.29–2.87; P = 0.87) for methylprednisolone (1 trial, 47 patients, and 26 deaths) [70].

There are conflicting results in the literature regarding the impact of IL-6 antagonist tocilizumab on mortality. However, in case of use in intensive care, it is vital to manage the infection control measures cautiously against secondary infection risk while its antiinflammatory effect is closely monitored [71]. There are many reports about anakinra, another antiinflammatory agent and IL-1 antagonist, suggesting that it positively affects mortality rates [72]. Depending on the effect of anakinra, which has a short duration of action on decreasing inflammatory markers, the treatment can be continued for 7–10 days.

Low-molecular-weight heparin is used for anticoagulant therapy, and the dose and duration of treatment should be determined according to the individual characteristics of the patient [73].

## 8. Conclusion

It is vital to provide appropriate basic intensive care support during the intensive care process of COVID-19–related critical patients and to closely monitor clinical/laboratory data during these treatments to reduce mortality. Especially in cases where respiratory support is provided, determining the transition timing to invasive ventilation according to the patient’s needs and performing it at the right time significantly affects patient outcomes.

During the pandemic, the measures taken to prevent the transmission of the disease, the medical treatments applied against the newly recognized viral infection, and the treatments performed in intensive care units have a crucial place in the fight against the virus.
